# Retraction Note: Selective Priming of Tumor Blood Vessels by Radiation Therapy Enhances Nanodrug Delivery

**DOI:** 10.1038/s41598-022-17483-7

**Published:** 2022-08-02

**Authors:** Sijumon Kunjachan, Shady Kotb, Robert Pola, Michal Pechar, Rajiv Kumar, Bijay Singh, Felix Gremse, Reza Taleeli, Florian Trichard, Vincent Motto-Ros, Lucie Sancey, Alexandre Detappe, Sayeda Yasmin-Karim, Andrea Protti, Ilanchezhian Shanmugam, Thomas Ireland, Tomas Etrych, Srinivas Sridhar, Olivier Tillement, Mike Makrigiorgos, Ross I. Berbeco

**Affiliations:** 1 Department of Radiation Oncology, Brigham and Women’s Hospital, Dana-Farber Cancer Institute and Harvard Medical School, Boston, MA United States; 2 Institut Lumière Matière, UMR 5306, Université Claude Bernard Lyon 1, CNRS, Villeurbanne, France; 3 Department of Physics, Nanomedicine Science and Technology Center, Northeastern University, Boston, MA United States; 4 Institute of Macromolecular Chemistry, Academy of Sciences of the Czech Republic, Heyrovsky Square 2, 16206 Prague 6, Czech Republic; 5 Experimental Molecular Imaging, University Hospital and Helmholtz Institute for Biomedical Engineering, RWTH Aachen University, Aachen, Germany; 6 Division of Medical Physics & Engineering, University of Texas Southwestern Medical Center, Texas, United States; 7 Institute for Advanced Biosciences, UMR 5309 Joint Research Center, UGA/INSERM U1209, CNRS, Grenoble, France; 8 Lurie Family Imaging Center, Department of Radiology, Dana-Farber Cancer Institute and Harvard Medical School, Boston, MA United States; 9 Department of Earth and Environmental Sciences, LA-ICP-MS and ICP-ES Laboratories, Boston University, Boston, MA United States

Retraction of: *Scientific Reports* 10.1038/s41598-019-50538-w, published online 01 November 2019

The Authors have retracted this Article.

Subsequent to the publication of a Correction [1], a number of errors were found in the archived data underlying Figure 2, including the duplication and mislabelling of images. The Authors therefore no longer have confidence in the reliability of the data presented.

Shady Kotb, Robert Pola, Michal Pechar, Rajiv Kumar, Bijay Singh, Reza Taleeli, Florian Trichard, Vincent Motto-Ros, Lucie Sancey, Alexandre Detappe, Sayeda Yasmin-Karim, Andrea Protti, Ilanchezhian Shanmugam, Thomas Ireland, Tomas Etrych, Srinivas Sridhar, Olivier Tillement, Mike Makrigiorgos, and Ross I. Berbeco agree with the retraction and its wording. Sijumon Kunjachan and Felix Gremse did not respond to the correspondence about this retraction.
